# Transcriptomic Analysis Implies That GA Regulates Sex Expression via Ethylene-Dependent and Ethylene-Independent Pathways in Cucumber (*Cucumis sativus* L.)

**DOI:** 10.3389/fpls.2017.00010

**Published:** 2017-01-19

**Authors:** Yan Zhang, Guiye Zhao, Yushun Li, Ning Mo, Jie Zhang, Yan Liang

**Affiliations:** ^1^College of Horticulture, Northwest A&F UniversityYangling, China; ^2^State Key Laboratory of Crop Stress Biology in Arid Region, Northwest A&F UniversityYangling, China

**Keywords:** cucumber, ethylene, gibberellin, sex expression, transcriptome

## Abstract

Sex differentiation of flower buds is an important developmental process that directly affects fruit yield of cucumber (*Cucumis sativus* L.). Plant hormones, such as gibberellins (GAs) and ethylene can promote development of male and female flowers, respectively, however, the regulatory mechanisms of GA-induced male flower formation and potential involvement of ethylene in this process still remain unknown. In this study, to unravel the genes and gene networks involved in GA-regulated cucumber sexual development, we performed high throughout RNA-Seq analyses that compared the transcriptomes of shoot tips between GA_3_ treated and untreated gynoecious cucumber plants. Results showed that GA_3_ application markedly induced male flowers but decreased ethylene production in shoot tips. Furthermore, the transcript levels of *M* (*CsACS2*) gene, ethylene receptor *CsETR1* and some ethylene-responsive transcription factors were dramatically changed after GA_3_ treatment, suggesting a potential involvement of ethylene in GA-regulated sex expression of cucumber. Interestingly, GA_3_ down-regulated transcript of a C-class floral homeotic gene, *CAG2*, indicating that GA may also influence cucumber sex determination through an ethylene-independent process. These results suggest a novel model for hormone-mediated sex differentiation and provide a theoretical basis for further dissection of the regulatory mechanism of male flower formation in cucumber.

**Statement:** We reveal that GA can regulate sex expression of cucumber via an ethylene-dependent manner, and the *M* (*CsACS2*), *CsETR1*, and *ERFs* are probably involved in this process. Moreover, *CAG2*, a C-class floral homeotic gene, may also participate in GA-modulated cucumber sex determination, but this pathway is ethylene-independent.

## Introduction

Cucumber (*Cucumis sativus* L.) is a typical monoecious plant with distinct male and female flowers, and has been served as a model system for studying physiological and molecular aspects of sex determination in plants (Malepszy and Niemirowicz-Szczytt, 1991; Bai and Xu, 2013). During the early stages of cucumber flower development, both stamen primordia and carpel primordia are initiated, however, sex differentiation occurs just after the hermaphroditic stage, subsequently, female or male flower is formed and developed through the selective developmental arrest of stamen or carpel, respectively (Bai et al., 2004).

Sex differentiation in cucumber is mainly determined by *F, M*, and *A* genes. Among them, *F* (*CsACS1G*) and *M* (*CsACS2*) genes encoding two ACC synthases (key enzymes in ethylene biosynthetic pathway) govern female sex expression in cucumber, and the *F* gene promotes female flower development (Trebitsh et al., 1997; Mibus and Tatlioglu, 2004; Knopf and Trebitsh, 2006), while the *M* gene inhibits stamen development in flower buds (Yamasaki et al., 2001, 2003; Saito et al., 2007; Li et al., 2009, 2012). In contrast, the *A* gene inhibits female flower development and facilitates male flower formation (Pierce and Wehner, 1990). The interaction of *F, M*, and *A* genes eventually determines various sexual phenotypes of cucumber.

In addition to genetic control, sex expression of cucumber can be affected by phytohormones, such as ethylene and GAs. Particularly, ethylene is considered as a potent sex hormone in cucumber that can induce formation of female flowers (Malepszy and Niemirowicz-Szczytt, 1991). Ethylene content in shoot tip of gynoecious cucumber is higher than that of monoecious plant (Rudich et al., 1972; Fujita and Fujieda, 1981; Trebitsh et al., 1987). Treatment with exogenous ethylene or ethylene-releasing reagent can increase the numbers of female and bisexual flowers in monoecious and andromonoecious lines, respectively (MacMurray and Miller, 1968; Iwahori et al., 1969). Until now, the molecular mechanism of ethylene-regulated sex determination of cucumber has been well understood. Except for the *F* and *M* genes, other ethylene biosynthetic genes, such as *CSACO2* and *CSACO3*, which encode ACC oxidases, are also involved in sex expression of cucumber, but the transcript levels of *CSACO2* and *CSACO3* in the shoot tips show a negative correlation with femaleness, indicating an existence of a feedback inhibition mechanism underlying such correlation (Kahana et al., 1999). Overexpression of *CsACO2*, driven by the *AP3* promoter, can arrest the stamen development by inducing chromatin condensation in *Arabidopsis* (Hao et al., 2003; Duan et al., 2008). Moreover, an ethylene receptor, *CsETR1*, has been demonstrated to play a key role in stamen arrest in female cucumber flowers through induction of DNA damage (Wang et al., 2010).

Gibberellins, one class of tetracyclic diterpenoid phytohormones, can promote the male tendency in cucumber. GA production in andromonoecious cucumber in higher than that in gynoecious and monoecious plants (Hemphill et al., 1972). Exogenous GA_3_ application can increase the ratio of maleness to femaleness in monoecious cucumber and induce the formation of male flowers in gynoecious plants (Wittwer and Bukovac, 1962; Pike and Peterson, 1969). In addition, GA signaling pathway is involved in stamen and anther development in hermaphroditic plants, such as *Arabidopsis* and rice (*Oryza sativa*) (Cheng et al., 2004; Fleet and Sun, 2005; Aya et al., 2009; Sun, 2010, 2011; Plackett et al., 2011; Song et al., 2013). In this pathway, GA first binds with *GID1* receptor and promotes the interaction between GID1 and DELLA proteins (repressors of GA signaling), leading to a rapid degradation of DELLA proteins by an ubiquitin-proteasome pathway, and the proteolysis of DELLA proteins releases their inhibitory effect on GA action and allows plant growth and development (Fleet and Sun, 2005; Murase et al., 2008; Harberd et al., 2009; Sun, 2010, 2011; Plackett et al., 2014). *GAMYB* is a positive regulator in GA signaling pathway and acts as an important downstream gene of DELLA proteins (Olszewski et al., 2002; Achard et al., 2004; Fleet and Sun, 2005). GA can induce *GAMYB* transcript through degradation of DELLA proteins, resulting in an enhanced flowering and anther development (Achard et al., 2004). In our previous studies, we identified two GA signaling genes, *CsGAIP* and *CsGAMYB1*, which belong to *DELLA* and *GAMYB* family, respectively. Both of them were predominantly expressed in the male specific organs during cucumber flower development. *CsGAIP* can inhibit stamen development through transcriptional repression of B class floral homeotic genes *APETALA3* (*AP3*) and *PISTILLATA* (*PI*) in *Arabidopsis* (Zhang et al., 2014a). However, whether *CsGAIP* is involved in GA-regulated sex determination in cucumber flowers is still unknown. Notably, *CsGAMYB1* can also mediate sex expression of cucumber. Knockdown of *CsGAMYB1* in cucumber results in decreased ratio of nodes with male to female flowers (Zhang et al., 2014b). Despite the current knowledge of GA-regulated sex expression of cucumber, the precise regulatory pathway in this complex process remains elusive.

Although both ethylene and GA can mediate sex expression of cucumber, their regulatory functions appear to be opposite. Atsmon and Tabbak (1979) interpreted that the GA-regulated sex differentiation has no effect on ethylene production, and there is a balance in the content of ethylene and GA in controlling the sex expression of cucumber. However, Yin and Quinn (1995) proposed a “one-hormone hypothesis” which posited that ethylene plays a dominant role in cucumber sex determination and GA may regulate the maleness through inhibiting ethylene production. However, our previous studies demonstrated that GA*-CsGAMYB1* signaling could regulate sex differentiation in cucumber through an ethylene-independent process (Zhang et al., 2014b). Therefore, a potential crosstalk between GA and ethylene pathways that determine sex expression in cucumber still remains unclear.

Besides, members of the MADS-box gene family can also regulate the sexual development in cucumber. *AGAMOUS* (*AG*), the C-class floral homeotic gene, specifies stamen and carpel identity (Lohmann and Weigel, 2002). There are three *AG* homologs in cucumber, *CAG1, CAG2*, and *CAG3*, in which *CAG1* and *CAG3* are expressed in both stamen and carpel, while *CAG2* is particularly restricted to the carpel. However, the expression levels of these three genes do not appear to be mediated by ethylene and GA (Kater et al., 1998; Perl-Treves et al., 1998). Moreover, *ERAF17*, an another MADS-box gene, can be induced by ethylene and may be involved in female flowers formation in cucumber (Ando et al., 2001).

In our study, in order to understand the genes and gene networks that may be involved in GA-modulated cucumber sex determination, we performed RNA-Seq analyses to compare the transcriptomes of shoot apices between GA_3_-treated and control gynoecious cucumber plants. GA_3_ application induced male flowers but reduced ethylene production in the shoot apices. Notably, GA-regulated sex differentiation was associated with the changes in transcript levels of *M* (*CsACS2*) gene, ethylene receptor *ETR1*, ethylene-responsive transcription factors, and *CAG2* (a C-class floral homeotic gene), suggesting a potential involvement of both ethylene-dependent and -independent processes in GA-mediated cucumber sexual development. Thus, our results built a foundation for dissecting the molecular mechanism of male flower development in cucumber.

## Materials and Methods

### Plant Materials and Growth Conditions

A gynoecious cucumber (*C. sativus* L.) line 13-3B was used in this study. The seeds were germinated on wet filter paper in a Petri dish at 28°C in dark overnight. Then the resulting seedlings were grown in a growth chamber under 16 h/8 h with 25°C/18°C in day/night, respectively. Upon two true-leaf stage, plants were transferred to a greenhouse in the experimental field of the Northwest A&F University. Pest control and water management were carried out according to standard practices.

### Exogenous GA_3_ Treatment

For male flowers induction in the gynoecious cucumber 13-3B, 1000 ppm GA_3_ (dissolved in 0.1% ethanol) or deionized water with 0.1% ethanol (Control) were applied by foliar spray for three times at 7 day intervals, starting when the first true leaf was approximately 2.5 cm in diameter. The sex of the flowers on each node of the main stem was recorded until anthesis of flowers on node 25.

In addition, ethylene production in shoot apices was measured after 7 days of the third GA_3_ treatment. And the RNA-Seq analyses were performed in shoot apices from the cucumber plants firstly treated with GA_3_ for 6 h, 12 h and the Control, respectively. GA_3_ was acquired from Sigma-Aldrich Chemical Co. (Shanghai, China).

### Quantification of Ethylene

The ethylene production was measured by gas chromatography as described previously with some modifications (Zhang et al., 2014b). In brief, the excised shoot apices from cucumber plants treated with exogenous GA_3_ and the Control were enclosed in 10 mL vessels after weighing and sealed with rubber stoppers. After incubation at 25°C for 16 h, 1 mL of head gas was withdrawn from each vessel using a syringe and injected into a gas chromatograph (GC-9A, Shimadzu, Japan) equipped with a hydrogen FID and an activated alumina column for the measurement of ethylene production. Standard ethylene gas was used for calibrating the instrument. Amount of ethylene was calculated per 1 g fresh weight and per hour.

### RNA Extraction and Quality Test

The shoot apices from the gynoecious cucumber plants were collected at 6 and 12 h after the first treatment with GA_3_ and the Control. Samples were immediately frozen in liquid nitrogen and stored at -80°C for RNA-Seq analyses. Total RNA was isolated using the RNA extraction kit (Promega, USA). RNA was checked by RNase-free agarose gel electrophoresis to avoid possible degradation and contamination, and then examined using the NanoPhotometer spectrophotometer (IMPLEN, Westlake Village, CA, USA) for RNA purity. RNA concentration and integrity were measured and assessed using the Qubit RNA Assay Kit in Qubit 2.0 Flurometer (Life Technologies, Carlsbad, CA, USA) and RNA Nano 6000 Assay Kit of the Bioanalyzer 2100 system (Agilent Technologies, Santa Clara, CA, USA), respectively.

### Digital Gene Expression (DGE) Library Construction and Sequencing

Digital gene expression libraries were constructed using the NEBNext Ultra Directional RNA Library Prep Kit for Illumina (NEB, Ispawich, USA) following instructions of manufacturer and six index codes were added to attribute sequences to various samples (Wang et al., 2009). Briefly, poly (A) mRNA was isolated from 3 μg total RNA using oligo-dT magnetic beads (Life Technologies, Carlsbad, CA, USA), and then broken into short fragments by adding fragmentation buffer. First-strand cDNA was synthesized using random hexamer-primed reverse transcription, followed by the synthesis of the second-strand cDNA using RNase H and DNA polymerase I. After adenylation of the 3′ ends of cDNA fragments, NEBNext adapter oligonucleotides were ligated to prepare for hybridization, and then the cDNA fragments were purified using AMPure XP system (Beckman Coulter, Beverly, MA, USA) to select the fragments of preferentially 150–200 bp in length. The size-selected, adaptor-ligated cDNA fragments were enriched using Phusion High-Fidelity DNA polymerase, Universal PCR primers and Index Primer in the PCR reaction. PCR products were purified with AMPure XP system and library quality was assessed using the Agilent Bioanalyzer 2100 system. At last, the cDNA libraries were sequenced on an Illumina HiSeq 4000 platform using the paired-end technology by Novogene Co. (Beijing, China).

### Bioinformatics Analysis of DGE Data

Raw reads were pre-processed to remove low quality sequences (there were more than 50% bases with quality lower than 20 in one sequence), reads with more than 5% N bases (bases unknown) and reads containing adaptor sequences. Then the clean reads were mapped to the cucumber genome (Chinese long) v2^1^ using TopHat (Huang et al., 2009; Trapnell et al., 2009), allowing up to one mismatch. Unigenes mapped by at least one read, in at least one sample, were identified for further analysis.

In this study, samples from three treatments (GA 6 h, GA 12 h, and Control) were prepared for genome-wide expression analyses. Two biological replicates were performed for each treatment, and thus six DGE libraries were sequenced. 44.28–60.29 million raw reads from each library were generated. After removal of low-quality tags and adapter sequences, 42.31–57.63 million high-quality clean reads with a total of 6.35–8.64G bases were obtained. Among these clean reads, the percentage of Q20 (base quality more than 20) and GC was 97.28–97.52% and 43.46–43.63%, respectively (**Table 1**). Furthermore, we clustered these clean reads into unique tags, which were mapped to the cucumber genome using TopHat (Huang et al., 2009; Trapnell et al., 2009). About 36.28–49.30 million clean reads (85.21–85.74% of total clean reads) from RNA-Seq data in the six libraries were mapped uniquely to the cucumber genome (**Table 1**).

**Table 1:** Summary of the transcriptome assembly.

Samples	Control_rep1	Control _rep2	GA 6 h_rep1	GA 6 h_rep2	GA 12 h_rep1	GA 12 h_rep2
Raw reads	50,991,998	56,788,784	60,288,014	58,393,488	44,280,996	52,072,848
Clean reads (%)	48,705,848 (95.52)	54,275,188 (95.57)	57,631,340 (95.59)	55,720,738 (95.42)	42,311,862 (95.55)	49,993,570 (96.01)
Clean bases	7.31G	8.14G	8.64G	8.36G	6.35G	7.50G
Q20 (%)	97.52	97.42	97.38	97.43	97.28	97.36
GC (%)	43.63	43.60	43.51	43.53	43.46	43.54
Mapped clean reads (%)	42,119,358 (86.48)	46,783,558 (86.20)	49,881,555 (86.55)	48,281,412 (86.65)	36,728,637 (86.80)	43,283,227 (86.58)
Unique mapped clean reads (%)	41,622,535 (85.46)	46,245,871 (85.21)	49,302,733 (85.55)	47,674,994 (85.56)	36,276,717 (85.74)	42,766,514 (85.54)

The R package edgeR used to identify the DGEs (Robinson et al., 2010). The expression level of each gene was calculated and normalized to FPKM. The FDR was used to determine the threshold of the P-value in multiple tests. In our study, the FDR < 0.05 and fold change > 2 were used as significance cut-offs of the expression differences.

Furthermore, GO enrichment analysis of DGEs was performed using the GOseq R package (Young et al., 2010). GO terms with corrected P-value < 0.05 were considered significantly enriched by differential expressed genes.

### Quantitative Real-Time PCR (qRT-PCR) Validation

Quantitative Real-Time PCR analyses were performed using the independent cucumber shoot apices in the same time point of GA_3_ application as those used for RNA-Seq. Total RNA was isolated using the RNA extraction kit (Promega, USA), and cDNA was synthesized using the PrimeScript RT reagent Kit (TaKaRa, China). qRT-PCR was carried out using SYBR Premix Ex Taq (TaKaRa, China) on an ABI 7500 Real-Time PCR System (Applied Biosystems, USA). The cucumber *α-TUBULIN* (*TUA)* gene was used as an internal control in analyzing gene expression. Three biological replicates were performed for each experiment. The gene specific primers for qRT-PCR are listed in Supplementary Table S5.

## Results

### Exogenous GA_3_ Induces Male Flowers Formation and Inhibits Ethylene Production in Gynoecious Cucumber

**FIGURE 1:**
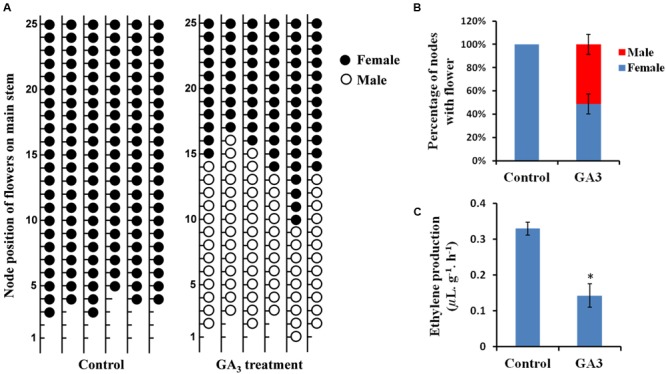
**Effects of exogenous GA_3_ on sex expression and ethylene production in gynoecious cucumber. (A)** Sex expression of the flowers on the first 25 nodes of the main stems in gynoecious cucumber plants treated with deionized water (with 0.1% ethanol) (Control) or 1000 ppm GA_3_. The black and white circles represent female and male flowers, respectively. Lower nodes without circle indicate the vegetative nodes. **(B)** The percentage of the nodes with female or male flowers up to the 25th node on the main stems of the Control and GA_3_ treatment lines. Values are the means ± SE from six independent plants. **(C)** Quantification of ethylene released from shoot tips in gynoecious cucumber plants at 7 days after treatment with GA_3_ and the Control. Six biological replicates were performed for this experiment. Vertical bars represent the standard errors. The asterisk indicates the significant difference (*P* < 0.01) between the Control and GA_3_ treatment lines by Duncan’s test.

To verify the effect of GA on sex expression of cucumber, a gynoecious cucumber line 13-3B was treated with GA_3_ and the sex of flowers on each node was recorded until anthesis of the flowers on node 25 of the main stems. As shown in **Figure 1**, there were no male flower nodes in the control plants, however, GA_3_ treatment induced male flowers in the gynoecious cucumber line (**Figure 1A**), accounting for 51.2% of nodes with male flowers (**Figure 1B**). Interestingly, formation of male flowers occurred mainly at the lower node positions as compared with the location of female flowers on the main stems (**Figure 1A**). These observations suggested that exogenous GA can promote male flowers formation in cucumber.

It is well known that ethylene can also control sex determination of cucumber (Malepszy and Niemirowicz-Szczytt, 1991; Bai and Xu, 2013). To assess potential involvement of ethylene in GA-regulated sex expression of cucumber, ethylene production in the shoot apices was measured in GA_3_-treated and control plants. As shown in **Figure 1C**, ethylene production was significantly decreased after GA_3_ treatment, suggesting that ethylene might function as a negative factor in GA-regulated male flower formation in cucumber.

### Identification of Differentially Expressed Genes (DEGs) in Shoot Apices from the Gynoecious Cucumber Plants Treated with GA_3_ and the Control

**FIGURE 2:**
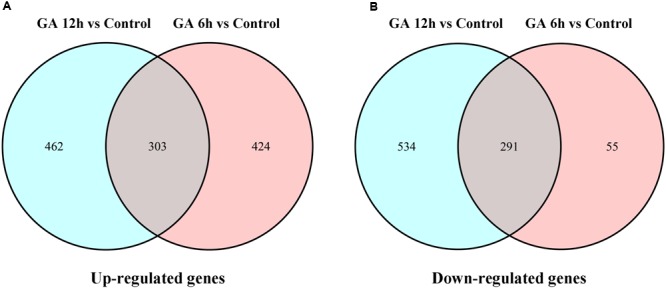
**Venn diagrams of DEGs that were significantly upregulated (A)** or downregulated **(B)** after 6 or 12 h of GA_3_ treatment.

To identify the genes and gene networks that are involved in GA-regulated cucumber sex expression, the genome-wide expression analyses were performed to compare the transcriptome profiles of the shoot apices between the gynoecious cucumber plants treated with GA_3_ for different time points (6 and 12 h) and the Control through the digital gene expression (DGE) approach (Eveland et al., 2010). Based on deep sequencing, 23,911 unigenes were collected in six libraries. Using fold change > 2 and FDR < 0.05 as the significance cut-offs, we identified 1073 DEGs including 727 up-regulated genes and 346 down-regulated genes after GA_3_ treatment for 6 h compared with the Control. And we also found that 1590 genes were differentially expressed, in which 765 genes were up-regulated and 825 genes were down-regulated after GA_3_ treatment for 12 h (**Figure 2**; Supplementary Tables S1 and S2). Moreover, 594 DEGs containing 303 up-regulated genes (**Figure 2A**) and 291 down-regulated genes (**Figure 2B**) were shared in the two sets of transcriptome comparisons.

### Verification of RNA-Seq Data by Quantitative Real Time RT-PCR Analyses

**FIGURE 3:**
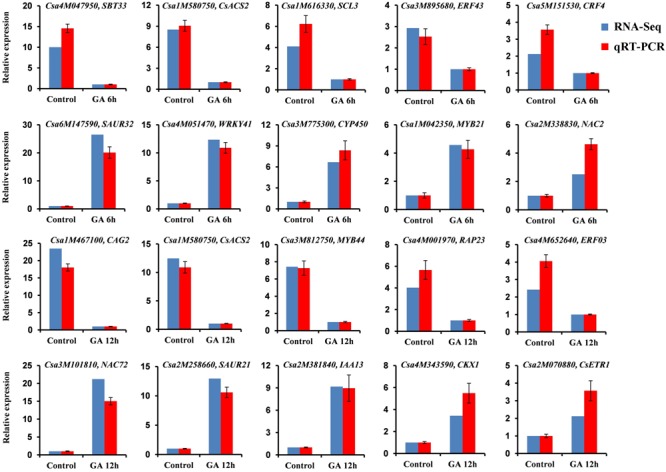
**qRT-PCR validation of DEGs identified by RNA-Seq.** Twenty DEGs including ten up-regulated genes and ten down-regulated genes from the two sets of transcriptome comparisons (GA 6 h vs. Control and GA 12 h vs. Control) were randomly selected for qRT-PCR confirmation. The blue and red bars represent RNA-Seq and qRT-PCR data, respectively. The cucumber *TUA* gene was used as an internal control, and these experiments were repeated with three biological samples. Error bars indicate the standard errors.

To validate the DEGs identified by RNA-Seq, we performed quantitative real time RT-PCR (qRT-PCR) assays using the independent cucumber shoot apices in the same time point after GA_3_ treatment as those used for RNA-Seq analysis. Twenty DEGs were randomly chosen for qRT-PCR analyses, in which 10 genes including five up-regulated genes and five down-regulated genes were from the set of GA 6 h vs. Control, and the other 10 genes containing five up-regulated genes and five down-regulated genes were from the set of GA 12 h vs. Control. As shown in **Figure 3**, all the 20 genes showed the similar expression patterns in the qRT-PCR analyses as those in the RNA-Seq data, although the particular values of fold-change were different. The Pearson correlation coefficient between qRT-PCR and RNA-Seq data was 0.975 (*P* = 3.5E-13), indicating that the RNA-Seq results were highly reliable.

### The *M* Gene Is Involved in GA-Regulated Sex Differentiation of Cucumber

Given that ethylene production was significantly decreased in the cucumber plants treated with GA_3_ (**Figure 1C**), we screened the ethylene biosynthetic genes in the DGEs. We found that the transcript of *M* (*CsACS2*) gene encoding an ACC synthase was inhibited in both sets of transcriptome comparisons (GA 6 h vs. Control and GA 12 h vs. Control) (**Table 2**), and the qRT-PCR verification displayed the same expression pattern (**Figure 3**). Since the *M* gene is believed to inhibit stamen development in flower buds (Saito et al., 2007; Li et al., 2009, 2012), we speculated that GA might release the inhibitory effect of the *M* gene and allow male flowers to develop in cucumber.

**Table 2:** List of differentially expressed ethylene biosynthetic genes identified by RNA-Seq in the shoot apices of GA_3_ treatment plants and the Control.

Gene ID	Gene annotation	Fold change (GA 6 h/Control)	FDR	Fold change (GA 12 h/Control)	FDR
Csa1M580750	*M (CsACS2, ACC Synthase 2*)	–8.53	5.33E-23	–12.47	9.94E-22
Csa6M160180	*CsACO1* (*ACC Oxidase 1*)	2.24	1.25E-06	2.56	5.59E-05
Csa6M421630	*CsACO3* (*ACC Oxidase 2*)	/	/	–2.07	6.92E-04

*The oblique line represents that the gene expression has no change in GA 6 h vs. Control.*

In addition, another two ethylene biosynthetic genes, *CsACO1* and *CsACO3* which encode two ACC oxidases, were also differently expressed in the shoot apices after GA_3_ treatment. *CsACO3* expression was dramatically down-regulated in the set of GA 12 h vs. Control, but not changed in GA 6 h vs. Control. However, there was an increase in the transcript level of *CsACO1* in both GA 6 h vs. Control and GA 12 h vs. Control, but the fold change was lower than that of *M* gene (**Table 2**). These results suggested that the decreased transcript levels of *M* and *CsACO3* might inhibit ethylene biosynthesis in the cucumber plants treated with GA_3_. Nonetheless, an increased expression of *CsACO1* following GA_3_ treatment was insufficient to rescue the effect of *M* and *CsACO3* on ethylene production.

### The Ethylene-Responsive Transcription Factors and Ethylene Receptor *CsETR1* Participate in GA-Modulated Cucumber Sex Expression

**FIGURE 4:**
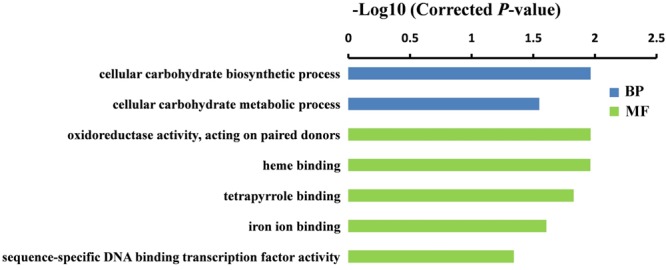
**Gene ontology (GO) terms that were significantly enriched in the DEGs after 6 h of GA_3_ treatment.** GO terms belong to biological processes (BP) and molecular functions (MFs) were shown in blue and green, respectively. GO terms were sorted based on corrected *P*-value, and the corrected *P*-value < 0.05 was used as the significance cut-off.

**FIGURE 5:**
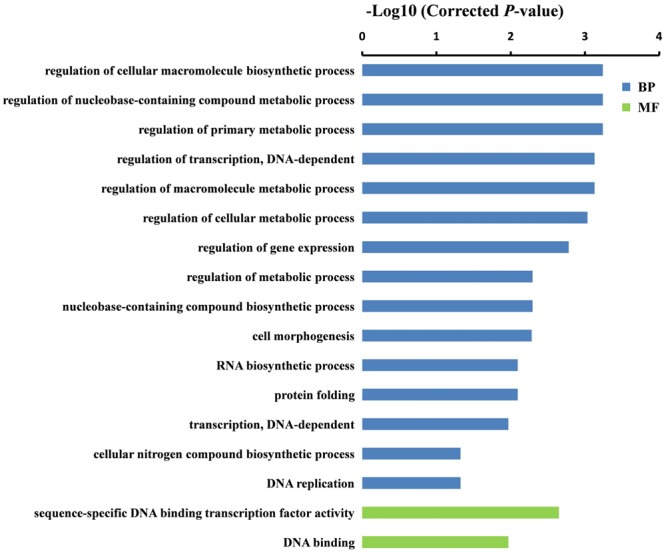
**Gene ontology terms that were significantly enriched in the DEGs after 12 h of GA_3_ treatment.** GO terms belong to BP and MFs were shown in blue and green, respectively. GO terms were sorted based on corrected *P*-value, and the corrected *P*-value < 0.05 was used as the significance cut-off.

To further understand the potential functions of DEGs identified by DGE, GO term enrichment analyses (Corrected *P*-value < 0.05) were carried out in both sets of RNA-Seq data. We found that the DEGs were markedly enriched in biological process and molecular function (MF) groups. For the biological process category, the most significantly enriched GO terms were “cellular carbohydrate biosynthetic process” (*P* = 1.1E-02) and “regulation of cellular macromolecule biosynthetic process” (*P* = 5.8E-04) in GA 6 h vs. Control and GA 12 h vs. Control groups, respectively (**Figures 4** and **5**). While five GO terms including “oxidoreductase activity, acting on paired donors” (*P* = 1.1E-02), heme binding (*P* = 1.1E-02), tetrapyrrole binding (*P* = 1.5E-02), iron ion binding (*P* = 2.5E-02), and “sequence-specific DNA binding transcription factor activity” (*P* = 4.6E-02) in the set of GA 6 h vs. Control (**Figure 4**) and two terms containing “sequence-specific DNA binding transcription factor activity” (*P* = 2.2E-03) and “DNA binding” (*P* = 1.1E-02) in the GA 12 h vs. Control group (**Figure 5**) were detected in the MF category. Furthermore, the transcription factors were dramatically enriched in the DGEs in both sets of data. Accordingly, many ethylene-responsive transcription factors (ERFs) including four in GA 6 h vs. Control (**Table 3** and Supplementary Table S3) and nine in GA 12 h vs. Control (**Table 4** and Supplementary Table S4) were identified to be down-regulated, consistent with the reduced ethylene production (**Figure 1C**). Among them, three genes, *ERF43* (*Csa3M895680*) and *CRF2s* (*Csa5M139630* and *Csa4M051360*), were shared in the two sets. These observations indicated that *ERFs* may be implicated in cucumber sex expression. Because the *ERFs* act as positive regulators in ethylene signal transduction pathway (Wang et al., 2002; Guo and Ecker, 2004; Prescott et al., 2016; Zhang et al., 2016), we speculated that the decreased ethylene production inhibited the expression of *ERFs*, followed by modulated sexual development in cucumber plants treated with GA_3_.

**Table 3:** List of selected ethylene signaling factors in the DEGs with enriched GO terms after GA_3_ treatment for 6 h.

Gene ID	Gene annotation	Fold change (GA 6 h/Control)	FDR
*Sequence-specific DNA binding transcription factor activity*			
Csa3M895680	*ERF43* (*Ethylene-responsive transcription factor ERF043*)	**–**2.93	1.08E-16
Csa5M139630	*CRF2* (*Ethylene-responsive transcription factor CRF2*)	**–**2.51	1.89E-02
Csa4M051360	*CRF2* (*Ethylene-responsive transcription factor CRF2*)	**–**2.23	2.66E-02
Csa5M151530	*CRF4* (*Ethylene-responsive transcription factor CRF4*)	**–**2.12	1.14E-03

**Table 4:** List of selected ethylene signaling factors and *AGAMOUS* (*AG*) homolog in the DEGs with enriched GO terms after GA_3_ treatment for 12 h.

Gene ID	Gene annotation	Fold change (GA 12 h/Control)	FDR
*equence-specific DNA binding transcription factor activity*			
Csa2M092800	*BBM2* (*AP2-like ethylene-responsive transcription factor BBM2*)	**–**∞	1.86E-02
Csa2M382540	*ERF03* (*Ethylene-responsive transcription factor ERF003*)	**–**3.44	3.98E-02
Csa2M382550	*ERF03* (*Ethylene-responsive transcription factor ERF003*)	**–**2.03	8.55E-05
Csa3M895680	*ERF43* (*Ethylene-responsive transcription factor ERF043*)	**–**3.06	1.93E-13
Csa4M001970	*RAP23* (*Ethylene-responsive transcription factor RAP2-3*)	**–**4.03	5.42E-10
Csa4M051360	*CRF2* (*Ethylene-responsive transcription factor CRF2*)	**–**2.44	3.40E-02
Csa4M192030	*RA211* (Ethylene-responsive transcription factor RAP2-11)	**–**3.87	4.03E-02
Csa4M652640	*ERF03* (*Ethylene-responsive transcription factor ERF003*)	**–**2.42	5.66E-03
Csa5M139630	*CRF2* (*Ethylene-responsive transcription factor CRF2*)	**–**3.02	9.74E-03
Csa1M467100	*CAG2* (*Floral homeotic protein AGAMOUS 2*)	**–**23.48	7.84E-03
*DNA binding*			
Csa2M070880	*CsETR1* (*Cucumber Ethylene receptor 1*)	2.12	6.99E-06

Interestingly, we also noticed that an ethylene receptor, *CsETR1*, was enriched in the GO term of “DNA binding” (**Figure 5**), and its expression was increased by 2.12-fold in shoot apices after GA_3_ treatment for 12 h (**Table 4** and Supplementary Table S4), but not changed in GA 6 h vs. Control group. While the qRT-PCR verification revealed the same expression pattern (**Figure 3**). *ETR1* is an important member of ethylene receptors family that acts as negative regulator in the ethylene signaling pathway (Wang et al., 2002; Guo and Ecker, 2004; Light et al., 2016; Prescott et al., 2016). Previous studied have confirmed that *CsETR1* plays a negative role in stamen arrest during development of flower buds in cucumber (Wang et al., 2010). In accordance with these findings, we speculated that up-regulated *CsETR1* may promote male flowers formation by alleviating stamen arrest in cucumber after GA_3_ treatment.

### GA May Restrain the Female Tendency via Transcriptional Inhibition on *CAG2* in Cucumber

Through GO term enrichment analyses, we further identified an *AG* (C-class floral homeotic gene) homolog *CAG2*, which was enriched in the “sequence-specific DNA binding transcription factor activity” group. *CAG2* is one of the three *AG* genes in cucumber that controls pistil development due to its specific expression in the carpel (Kater et al., 1998; Perl-Treves et al., 1998). We found that the transcript level of *CAG2* was significantly decreased by 23.48-fold in shoot apices after 12 h of GA_3_ treatment (**Table 4** and Supplementary Table S4), and the qRT-PCR assay showed the same expression pattern (**Figure 3**). Our data implied that GA may restrain the femaleness via inhibiting the *CAG2* expression in cucumber.

## Discussion

**FIGURE 6:**
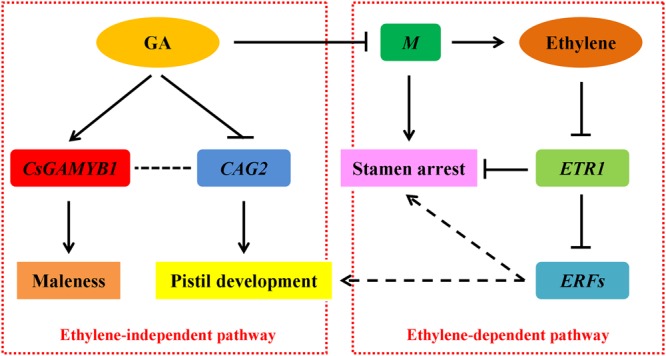
**A proposed model showing GA-regulated sex expression in cucumber.** GA can promote male flowers formation via ethylene-dependent (right) and ethylene-independent (left) pathways. Arrows and T-bars indicate positive and negative effects, respectively. The solid lines define proved regulations, whereas the dotted lines represent proposed regulations.

Sex differentiation of flower buds is an important developmental process that directly affects the product yield in cucumber. In addition to genetic control, sex expression can be modified by plant hormones and environmental conditions. (Malepszy and Niemirowicz-Szczytt, 1991). Among various plant hormones, ethylene can induce female flowers formation (MacMurray and Miller, 1968; Iwahori et al., 1969), and the underlying molecular mechanism has been widely documented (Yamasaki et al., 2001, 2003; Mibus and Tatlioglu, 2004; Knopf and Trebitsh, 2006; Saito et al., 2007; Li et al., 2009, 2012; Wang et al., 2010). GA can promote male flowers development (Wittwer and Bukovac, 1962; Pike and Peterson, 1969), but the regulatory pathway remains elusive. In addition, a potential crosstalk between GA and ethylene in controlling sex determination of cucumber still remains disputed. In this study, through genome-wide expression analyses, we showed that GA may promote cucumber maleness via an ethylene-dependent pathway by altering expression of the *M* (*CsACS2*) gene, ethylene receptor *CsETR1* and ethylene-responsive transcription factors. Nevertheless, we also found that GA may also restrain femaleness through an ethylene-independent pathway regulating *CAG2*, a C-class floral homeotic gene (**Figure 6**).

### GA May Promote Male Tendency via an Ethylene-Dependent Pathway in Cucumber

Upon exogenous GA_3_ treatment in the gynoecious cucumber line 13-3B, the male flowers were markedly induced, meanwhile, ethylene production in the shoot apices was significantly decreased (**Figure 1**) due to collaborative regulation by three ethylene biosynthetic genes such as *M* (*CsACS2*), *CsACO1*, and *CsACO3*. Notably, *M* and *CsACO1* were significantly down-regulated and up-regulated in both sets of transcriptome comparisons, respectively, however, *CsACO3* transcript was only decreased in GA 12 h vs. Control group (**Figure 3**; **Table 2**). Since both *CsACO1* and *CsACO3* encode ACC oxidases, and they showed opposite expression patterns and similar fold changes in RNA-Seq data (**Table 2**), we speculated that interplay between up-regulation of *CsACO1* and down-regulation of *CsACO3* offset the ACC oxidase activity in shoot apices after 12 h of GA_3_ treatment. This implied that the reduced *M* transcript might play major role in inhibiting ethylene biosynthesis. Given that the *M* gene can directly inhibit stamen development in cucumber flower buds (Saito et al., 2007; Li et al., 2009, 2012), our data revealed that GA might release the inhibitory effect of the *M* gene on stamen arrest and restrain ethylene production, by down-regulating the *M* gene expression (**Figure 6**, right panel).

In ethylene signal transduction pathway, the receptors such as ETRs function as negative regulators, while ERFs, downstream components of receptors, act as positive transcription factors (Wang et al., 2002; Guo and Ecker, 2004; Light et al., 2016; Prescott et al., 2016; Zhang et al., 2016). RNA-Seq data displayed that the ethylene receptor *CsETR1* in cucumber, which is thought to be a negative regulatory factor in stamen arrest of flower buds (Wang et al., 2010), was markedly up-regulated after 12 h of GA_3_ treatment (**Figure 3**; **Table 4** and Supplementary Table S4). Up-regulation of *CsETR1* occurred at relatively later time point than down-regulation of the *M* gene (**Table 2**), suggesting that the increased *CsETR1* transcript was not directly caused by GA_3_ rather by reduced ethylene production. Moreover, the expression levels of some *ERFs* were dramatically decreased after GA_3_ treatment (**Tables 3** and **4**, Supplementary Tables S3 and S4), and they were probably involved in cucumber sex expression through inhibiting maleness or promoting femaleness. However, this understanding was based on bioinformatics analysis, the precise roles of *ERFs* on cucumber flower development remained unclear and should be verified in future studies using advanced physiological and molecular techniques. These observations indicated that increased *CsETR1* expression may stimulate male tendency through direct inhibition in stamen arrest or down-regulation on the *ERFs* transcript in cucumber after GA_3_ treatment (**Figure 6**, right panel).

Furthermore, despite we have shown that GA can regulate cucumber sex expression in cooperation with ethylene, how GA modulated ethylene and which genes participated in this process were unknown. Given that the DELLA proteins are central repressors of GA responses (Sun, 2010, 2011; Plackett et al., 2014), and accumulating evidence suggested that DELLA proteins play important roles in ethylene-mediated plant growth and development processes through interactions with some regulatory factors in ethylene signaling pathway, such as CTR1 (CONSTITUTIVE TRIPLE RESPONSE1), EIN3/EIL1 (ETHYLENE INSENSITIVE 3/EIN3-LIKE 1), RAP 2.3 (RELATED TO APETALA 2.3), and ERF11 (ETHYLENE RESPONSE FACTOR 11) (Achard et al., 2003, 2007; Pierik et al., 2009; An et al., 2012; Luo et al., 2013; Marín-de la Rosa et al., 2014; Zhou et al., 2016). Therefore, we proposed that DELLA proteins may be involved in the sex differentiation of cucumber coupled with GA and ethylene in a collaborative regulation at the protein level, since the expressions of four *DELLA* homologs in cucumber, *CsGAIP, CsGAI1, CsGAI2*, and *CsGAI3* (Zhang et al., 2014a), had no change after GA_3_ treatment (Supplementary Tables S1 and S2).

In addition, GA and ethylene can also cooperatively regulate other aspects of plant growth and development. For example, GA promoted apical hook development of *Arabidopsis*, in part through transcriptional regulation of several genes in ethylene biosynthetic pathway mediated by DELLA proteins (Gallego-Bartolome et al., 2011). In this process, GA induced ethylene production, that was opposite to our results in cucumber sex expression. We speculated that this distinction may be due to different roles of hormones in various plant developmental processes. In fact, GA and ethylene showed similar functions in hook development, but they may play opposite roles in sex determination, thus, the regulatory mechanisms were different.

### GA May Inhibit Femaleness or Induce Maleness of Cucumber via an Ethylene-Independent Pathway

*AGAMOUS*, the C-class floral homeotic gene, belongs to the MADS-box family. In *Arabidopsis*, GA can induce the expression of *AG* gene (Yu et al., 2004). However, our data provided a novel point that an *AG* homolog *CAG2* in cucumber was down-regulated upon GA_3_ treatment (**Figure 3**; **Table 4** and Supplementary Table S4). This distinction may be due to different abilities of these two genes to induce reproductive organ fate, in which, *AG* in *Arabidopsis* controls stamen and carpel development (Lohmann and Weigel, 2002), but *CAG2* is particularly restricted to the carpel (Kater et al., 1998; Perl-Treves et al., 1998). Previous studies showed that *CAG2* transcripts were not mediated by ethylene (Perl-Treves et al., 1998), hence, we speculated that GA probably suppressed pistil development through inhibiting the *CAG2* expression, thereby allowing male flowers to develop, and ethylene was not involved in this process. Moreover, in our previous study, we demonstrated that transcript of *CsGAMYB1* was upregulated by GA_3_ treatment in male flower buds and silencing of *CsGAMYB1* could suppress masculinization of cucumber, but the ethylene production and expression of *F* and *M* genes were not changed in the *CsGAMYB1*-RNAi lines (Zhang et al., 2014b). These observations suggested that GA can also regulate sex expression of cucumber via an ethylene-independent pathway (**Figure 6**, left panel). However, the relationship between *CAG2* and *CsGAMYB1* regulations remains obscure and that should be verified in further studies.

Besides, DELLA proteins can down-regulate the expression of floral homeotic genes, *AP3, PI* and *AG*, subsequently inhibit flower development in *Arabidopsis* (Yu et al., 2004). *CsGAIP*, a DELLA homolog in cucumber, may restrain staminate development through transcriptional repression of *AP3* and *PI* in *Arabidopsis* (Zhang et al., 2014a). However, the expression levels of *AP3* and *PI* were not changed after GA_3_ treatment (Supplementary Tables S1 and S2), indicating that they did not participate in GA-regulated male tendency. Accordingly, there may be a regulatory relationship between DELLA proteins and floral homeotic gene *CAG2* during GA-modulated sexual development in cucumber, however, the mechanism would be possibly different from that of *Arabidopsis*.

In summary, our data revealed a novel viewpoint that GA might control sex differentiation of cucumber via both ethylene-dependent and ethylene-independent pathways, and DELLA proteins were likely to be involved in both processes. However, this model was proposed by bioinformatics data, therefore, elucidation of the critical roles of DELLA proteins in flower development by cucumber transformation, and identification of the relationships among DELLAs and ethylene regulatory factors, GA-DELLA*-CsGAMYB1* signaling and *CAG2* gene, will shed new light on the molecular details of GA-regulated sex expression in cucumber.

### Evolution of Unisexual Flower in Cucumber and Potential Involvement of Hormones

Generally, typical unisexual flowers have two morphological types. The type I is unisexual by abortion. Initiation of stamen and pistil occurs in all flowers, followed by the developmental arrest in one or another organ. The type II is unisexual from inception. Only stamen or pistil is initiated and it does not go through a hermaphroditic stage (Lebel-Hardenack and Grant, 1997; Ainsworth, 2000; Mitchell and Diggle, 2005). Now, it is believed that the morphology of cucumber flowers belongs to the type I (Bai et al., 2004), but its evolutionary mechanism is largely unknown. Bai and Xu proposed a “miR initiative” hypothesis (Bai and Xu, 2013), where they speculated that unisexual cucumber flowers are evolved from a hermaphrodite ancestor. The first step in the evolutionary process might be the miRNA-regulated arrest of ovary development, and this predication is based on the altered expression of miRNAs, such as miR396a, 156b, 159a, 171b, and 166a, in male flowers (Bai et al., 2004; Sun et al., 2010). And this event leads to environment-dependent andromonoecy which has no progeny. Then, the *M* gene is recruited. On the one hand, the *M* gene promotes ethylene biosynthesis, resulting in the rescue of ovary development for seed set, because the ethylene might regulate the miRNA production. On the other hand, the *M* gene inhibits stamen development to avoid self-pollination and maintain cross-pollination. So, the monoecious genotype is generated through the cooption of the *M* gene. The andromonoecious genotype is produced by the loss-of-function *m* gene, which is regarded as a reverted point mutation. Further, the *F* gene is coopted and generate the gynoecious genotype (Sun et al., 2010).

Until now, a potential role of GA in unisexual flower evolution of cucumber has not been reported. But based on the possible function of *M* gene on evolutionary development of cucumber flower and the effect of GA on the transcript of *M* gene (**Table 2**), we speculated that GA might be involved in the process of cucumber flower evolution through interaction with ethylene. In addition, previous studies showed that GA signaling system can regulate anther development by modulation of miR159/GAMYB (Achard et al., 2004). In this pathway, miR159 acts as a post-transcriptional regulator of *GAMYB* transcript levels. GA relieves the DELLA repression of GAMYB, which is mediated by the GA activation of miR159. As mentioned above, miR159 is likely to participate in the arrest of ovary development in the evolutionary process of cucumber flower. And the *GAMYB* homolog *CsGAMYB1* can regulate cucumber sex expression via an ethylene-independent pathway (Zhang et al., 2014b). These observations further revealed the possible involvement of GA in unisexual flower evolution of cucumber, but this process might be dependent on miR159 and GAMYB, and have no relationship with ethylene. Finally, it is worth noting that these viewpoints are built on the basis of the “miR initiative” hypothesis and needed to be tested in further work.

## Author Contributions

YZ and YaL designed the experiments. YZ, GZ, and NM performed the experiments. YZ, YuL, and JZ analyzed the data. YZ wrote the paper along with YaL. All authors reviewed the manuscript.

## Conflict of Interest Statement

The authors declare that the research was conducted in the absence of any commercial or financial relationships that could be construed as a potential conflict of interest.

## Footnotes

1 http://www.icugi.org/

## Abbreviations

ACC: 1-aminocyclopropane-1-carboxylate
DEG: differentially expressed gene
DGE: digital gene expression
FDR: false discovery rate
FID: flame ionization detector
FPKM: fragments per kilobase of transcript sequence per millions base pairs sequenced
GA: gibberellin
GO: gene ontology
qRT-PCR: quantitative real-time PCR

## References

[B1] Achard P. Baghour M. Chapple A. Hedden P. Van Der Straeten D. Genschik P. (2007). The plant stress hormone ethylene controls floral transition via DELLA-dependent regulation of floral meristem-identity genes. *Proc. Natl. Acad. Sci. U.S.A.* 104 6484–6489. 10.1073/pnas.0610717104 17389366 PMC1851083

[B2] Achard P. Herr A. Baulcombe D. C. Harberd N. P. (2004). Modulation of floral development by a gibberellin-regulated microRNA. *Development* 131 3357–3365. 10.1242/dev.01206 15226253

[B3] Achard P. Vriezen W. H. Van Der Straeten D. Harberd N. P. (2003). Ethylene regulates *arabidopsis* development via the modulation of DELLA protein growth repressor function. *Plant Cell* 15 2816–2825. 10.1105/tpc.015685 14615596 PMC282807

[B4] An F. Zhang X. Zhu Z. Ji Y. He W. Jiang Z. (2012). Coordinated regulation of apical hook development by gibberellins and ethylene in etiolated *Arabidopsis* seedlings. *Cell Res.* 22 915–927. 10.1038/cr.2012.29 22349459 PMC3343656

[B5] Ando S. Sato Y. Kamachi S. Sakai S. (2001). Isolation of a MADS-box gene (ERAF17) and correlation of its expression with the induction of formation of female flowers by ethylene in cucumber plants (*Cucumis sativus* L.). *Planta* 213 943–952. 10.1007/s004250100571 11722131

[B6] Ainsworth C. (2000). Boys and girls come out to play: the molecular biology of dioecious plants. *Ann. Bot.* 86 211–221. 10.1006/anbo.2000.1201

[B7] Atsmon D. Tabbak C. (1979). Comparative effects of gibberellin, silver nitrate and aminoethoxyvinyl glycine on sexual tendency and ethylene evolution in the cucumber plant (*Cucumis sativus* L.). *Plant Cell Physiol.* 20 1547–1555.

[B8] Aya K. Ueguchi-Tanaka M. Kondo M. Hamada K. Yano K. Nishimura M. (2009). Gibberellin modulates anther development in rice via the transcriptional regulation of GAMYB. *Plant Cell* 21 1453–1472. 10.1105/tpc.108.062935 19454733 PMC2700530

[B9] Bai S. L. Peng Y. B. Cui J. X. Gu H. T. Xu L. Y. Li Y. Q. (2004). Developmental analyses reveal early arrests of the spore-bearing parts of reproductive organs in unisexual flowers of cucumber (*Cucumis sativus* L.). *Planta* 220 230–240. 10.1007/s00425-004-1342-2 15290297

[B10] Bai S. N. Xu Z. H. (2013). Unisexual cucumber flowers, sex and sex differentiation. *Int. Rev. Cell Mol. Biol.* 304 1–55. 10.1016/B978-0-12-407696-9.00001-4 23809434

[B11] Cheng H. Qin L. Lee S. Fu X. Richards D. E. Cao D. (2004). Gibberellin regulates *Arabidopsis* floral development via suppression of DELLA protein function. *Development* 131 1055–1064. 10.1242/dev.00992 14973286

[B12] Duan Q. H. Wang D. H. Xu Z. H. Bai S. N. (2008). Stamen development in *Arabidopsis* is arrested by organ-specific overexpression of a cucumber ethylene synthesis gene CsACO2. *Planta* 228 537–543. 10.1007/s00425-008-0756-7 18506477

[B13] Eveland A. L. Satoh-Nagasawa N. Goldshmidt A. Meyer S. Beatty M. Sakai H. (2010). Digital gene expression signatures for maize development. *Plant Physiol.* 154 1024–1039. 10.1104/pp.110.159673 20833728 PMC2971585

[B14] Fleet C. M. Sun T. P. (2005). A DELLAcate balance: the role of gibberellin in plant morphogenesis. *Curr. Opin. Plant Biol.* 8 77–85. 10.1016/j.pbi.2004.11.015 15653404

[B15] Fujita Y. Fujieda K. (1981). Relation between sex expression types and cotyledon etiolation of cucumber in vitro. I. on the role of ethylene evolved from seedlings. *Plant Cell Physiol.* 22 667–674.

[B16] Gallego-Bartolome J. Arana M. V. Vandenbussche F. Zadnikova P. Minguet E. G. Guardiola V. (2011). Hierarchy of hormone action controlling apical hook development in *Arabidopsis*. *Plant J.* 67 622–634. 10.1111/j.1365-313X.2011.04621.x 21535259

[B17] Guo H. Ecker J. R. (2004). The ethylene signaling pathway: new insights. *Curr. Opin. Plant Biol.* 7 40–49. 10.1016/j.pbi.2003.11.011 14732440

[B18] Hao Y. J. Wang D. H. Peng Y. B. Bai S. L. Xu L. Y. Li Y. Q. (2003). DNA damage in the early primordial anther is closely correlated with stamen arrest in the female flower of cucumber (*Cucumis sativus* L.). *Planta* 217 888–895. 10.1007/s00425-003-1064-x 12898252

[B19] Harberd N. P. Belfield E. Yasumura Y. (2009). The angiosperm gibberellin-GID1-DELLA growth regulatory mechanism: how an “inhibitor of an inhibitor” enables flexible response to fluctuating environments. *Plant Cell* 21 1328–1339. 10.1105/tpc.109.066969 19470587 PMC2700538

[B20] Hemphill D. D. Baker L. R. Sell H. M. (1972). Different sex phenotypes of *Cucumis sativus* L. and C. melo L. and their endogenous gibberellin activity. *Euphytica* 21 285–291. 10.1007/BF00036769

[B21] Huang S. Li R. Zhang Z. Li L. Gu X. Fan W. (2009). The genome of the cucumber, *Cucumis sativus* L. *Nat. Genet.* 41 1275–1281. 10.1038/ng.475 19881527

[B22] Iwahori S. Lyons J. M. William L. S. (1969). Induced femaleness in cucumber by 2-chloroethanephosphonic acid. *Nature* 222 271–272. 10.1038/222271a0 5778393

[B23] Kahana A. Silberstein L. Kessler N. Goldstein R. S. Perl-Treves R. (1999). Expression of ACC oxidase genes differs among sex genotypes and sex phases in cucumber. *Plant Mol. Biol.* 41 517–528. 10.1023/A:1006343707567 10608661

[B24] Kater M. M. Colombo L. Franken J. Busscher M. Masiero S. Van Lookeren Campagne M. M. (1998). Multiple AGAMOUS homologs from cucumber and petunia differ in their ability to induce reproductive organ fate. *Plant Cell* 10 171–182. 10.1105/tpc.10.2.171 9490741 PMC143982

[B25] Knopf R. R. Trebitsh T. (2006). The female-specific Cs-ACS1G gene of cucumber. A case of gene duplication and recombination between the non-sex-specific 1-aminocyclopropane-1-carboxylate synthase gene and a branched-chain amino acid transaminase gene. *Plant Cell Physiol.* 47 1217–1228. 10.1093/pcp/pcj092 16887844

[B26] Lebel-Hardenack S. Grant S. R. (1997). Genetics of sex determination in flowering plants. *Trends Plant Sci.* 2 130–136. 10.1016/S1360-1385(97)01012-1

[B27] Li Z. Huang S. Liu S. Pan J. Zhang Z. Tao Q. (2009). Molecular isolation of the M gene suggests that a conserved-residue conversion induces the formation of bisexual flowers in cucumber plants. *Genetics* 182 1381–1385. 10.1534/genetics.109.104737 19474195 PMC2728875

[B28] Li Z. Wang S. Tao Q. Pan J. Si L. Gong Z. (2012). A putative positive feedback regulation mechanism in CsACS2 expression suggests a modified model for sex determination in cucumber (*Cucumis sativus* L.). *J. Exp. Bot.* 63 4475–4484. 10.1093/jxb/ers123 22577183 PMC3421985

[B29] Light K. M. Wisniewski J. A. Vinyard W. A. Kieber-Emmons M. T. (2016). Perception of the plant hormone ethylene: known-knowns and known-unknowns. *J. Biol. Inorg. Chem.* 21 715–728. 10.1007/s00775-016-1378-3 27456611

[B30] Lohmann J. U. Weigel D. (2002). Building beauty: the genetic control of floral patterning. *Dev. Cell* 2 135–142. 10.1016/S1534-5807(02)00122-3 11832239

[B31] Luo J. Ma N. Pei H. Chen J. Li J. Gao J. (2013). A DELLA gene, RhGAI1, is a direct target of EIN3 and mediates ethylene-regulated rose petal cell expansion via repressing the expression of RhCesA2. *J. Exp. Bot.* 64 5075–5084. 10.1093/jxb/ert296 24014864 PMC3830487

[B32] MacMurray A. L. Miller C. M. (1968). Cucumber sex expression modified by 2-chloroethanephosphonic acid. *Science* 162 1397–1398. 10.1126/science.162.3860.1397 5699656

[B33] Malepszy S. Niemirowicz-Szczytt K. (1991). Sex determination in cucumber (*Cucumis sativus*) as a model system for molecular biology. *Plant Sci.* 80 39–47. 10.1016/0168-9452(91)90271-9

[B34] Marín-de la Rosa N. Sotillo B. Miskolczi P. Gibbs D. J. Vicente J. Carbonero P. (2014). Large-scale identification of gibberellin-related transcription factors defines group VII ETHYLENE RESPONSE FACTORS as functional DELLA partners. *Plant Physiol.* 166 1022–1032. 10.1104/pp.114.244723 25118255 PMC4213073

[B35] Mibus H. Tatlioglu T. (2004). Molecular characterization and isolation of the F/f gene for femaleness in cucumber (*Cucumis sativus* L.). *Theor. Appl. Genet.* 109 1669–1676. 10.1007/s00122-004-1793-7 15490106

[B36] Mitchell C. H. Diggle P. K. (2005). The evolution of unisexual flowers: morphological and functional convergence results from diverse developmental transitions. *Am. J. Bot.* 92 1068–1076. 10.3732/ajb.92.7.1068 21646128

[B37] Murase K. Hirano Y. Sun T. P. Hakoshima T. (2008). Gibberellin-induced DELLA recognition by the gibberellin receptor GID1. *Nature* 456 459–463. 10.1038/nature07519 19037309

[B38] Olszewski N. Sun T. P. Gubler F. (2002). Gibberellin signaling: biosynthesis, catabolism, and response pathways. *Plant Cell* 14(Suppl.) S61–S80. 12045270 10.1105/tpc.010476 PMC151248

[B39] Perl-Treves R. Kahana A. Rosenmann N. Xiang Y. Silberstein L. (1998). Expression of multiple AGAMOUS-like genes in male and female flowers of cucumber (*Cucumis sativus* L.). *Plant Cell Physiol.* 39 701–710. 10.1093/oxfordjournals.pcp.a029424 9729894

[B40] Pierce L. K. Wehner T. C. (1990). Review of genes and linkage groups in cucumber. *HortScience* 25 605–615.

[B41] Pierik R. Djakovic-Petrovic T. Keuskamp D. H. de Wit M. Voesenek L. A. (2009). Auxin and ethylene regulate elongation responses to neighbor proximity signals independent of gibberellin and della proteins in *Arabidopsis*. *Plant Physiol.* 149 1701–1712. 10.1104/pp.108.133496 19211699 PMC2663759

[B42] Pike L. M. Peterson C. E. (1969). Gibberellin A4/A7, for induction of staminate flowers on the gynoecious cucumber (*Cucumis sativus* L.). *Euphytica* 18 106–109.

[B43] Plackett A. R. Ferguson A. C. Powers S. J. Wanchoo-Kohli A. Phillips A. L. Wilson Z. A. (2014). DELLA activity is required for successful pollen development in the Columbia ecotype of *Arabidopsis*. *New Phytol.* 201 825–836. 10.1111/nph.12571 24400898 PMC4291109

[B44] Plackett A. R. Thomas S. G. Wilson Z. A. Hedden P. (2011). Gibberellin control of stamen development: a fertile field. *Trends Plant Sci.* 16 568–578. 10.1016/j.tplants.2011.06.007 21824801

[B45] Prescott A. M. McCollough F. W. Eldreth B. L. Binder B. M. Abel S. M. (2016). Analysis of network topologies underlying ethylene growth response kinetics. *Front. Plant Sci.* 7:1308 10.3389/fpls.2016.01308 PMC5003821 27625669

[B46] Robinson M. D. McCarthy D. J. Smyth G. K. (2010). edgeR: a Bioconductor package for differential expression analysis of digital gene expression data. *Bioinformatics* 26 139–140. 10.1093/bioinformatics/btp616 19910308 PMC2796818

[B47] Rudich J. Halevy A. H. Kedar N. (1972). The level of phytohormones in monoecious and gynoecious cucumbers as affected by photoperiod and ethephon. *Plant Physiol.* 50 585–590. 10.1104/pp.50.5.585 16658222 PMC366195

[B48] Saito S. Fujii N. Miyazawa Y. Yamasaki S. Matsuura S. Mizusawa H. (2007). Correlation between development of female flower buds and expression of the CS-ACS2 gene in cucumber plants. *J. Exp. Bot.* 58 2897–2907. 10.1093/jxb/erm141 17630291

[B49] Song S. Qi T. Huang H. Xie D. (2013). Regulation of stamen development by coordinated actions of jasmonate, auxin, and gibberellin in *Arabidopsis*. *Mol. Plant* 6 1065–1073. 10.1093/mp/sst054 23543439

[B50] Sun J. J. Li F. Li X. Liu X. C. Rao G. Y. Luo J. C. (2010). Why is ethylene involved in selective promotion of female flower development in cucumber? *Plant Signal. Behav.* 5 1052–1056. 10.4161/psb.5.8.12411 20657187 PMC3115196

[B51] Sun T. P. (2010). Gibberellin-GID1-DELLA: a pivotal regulatory module for plant growth and development. *Plant Physiol.* 154 567–570. 10.1104/pp.110.161554 20921186 PMC2949019

[B52] Sun T. P. (2011). The molecular mechanism and evolution of the GA-GID1-DELLA signaling module in plants. *Curr. Biol.* 21 R338–R345. 10.1016/j.cub.2011.02.036 21549956

[B53] Trapnell C. Pachter L. Salzberg S. L. (2009). TopHat: discovering splice junctions with RNA-Seq. *Bioinformatics* 25 1105–1111. 10.1093/bioinformatics/btp120 19289445 PMC2672628

[B54] Trebitsh T. Rudich J. Riov J. (1987). Auxin, biosynthesis of ethylene and sex expression in cucumber (*Cucumis sativus*). *Plant Growth Regul.* 5 105–113. 10.1007/BF00024738

[B55] Trebitsh T. Staub J. E. O’Neill S. D. (1997). Identification of a 1-aminocyclopropane-1-carboxylic acid synthase gene linked to the female (F) locus that enhances female sex expression in cucumber. *Plant Physiol.* 113 987–995. 10.1104/pp.113.3.987 9085580 PMC158220

[B56] Wang D. H. Li F. Duan Q. H. Han T. Xu Z. H. Bai S. N. (2010). Ethylene perception is involved in female cucumber flower development. *Plant J.* 61 862–872. 10.1111/j.1365-313X.2009.04114.x 20030751

[B57] Wang L. C. Li H. Joseph R. E. (2002). Ethylene biosynthesis and signaling networks. *Plant Cell* 14(Suppl. 14) S131–S151. 12045274 10.1105/tpc.001768 PMC151252

[B58] Wang Z. Gerstein M. Snyder M. (2009). RNA-Seq: a revolutionary tool for transcriptomics. *Nat. Rev. Genet.* 10 57–63. 10.1038/nrg2484 19015660 PMC2949280

[B59] Wittwer S. H. Bukovac M. I. (1962). Staminate flower formation on gynoecious cucumber as influenced by the various gibberellins. *Naturwissenshaften* 49 305–306. 10.1007/BF00622719

[B60] Yamasaki S. Fujii N. Matsuura S. Mizusawa H. Takahashi H. (2001). The M locus and ethylene-controlled sex determination in andromonoecious cucumber plants. *Plant Cell Physiol.* 42 608–619. 10.1093/pcp/pce076 11427680

[B61] Yamasaki S. Fujii N. Takahashi H. (2003). Characterization of ethylene effects on sex determination in cucumber plants. *Sex. Plant Reprod.* 16 103–111. 10.1007/s00497-003-0183-7

[B62] Yin T. Quinn J. A. (1995). Tests of a mechanistic model of one hormone regulating both sexes in *Cucumis sativus* (Cucurbitaceae). *Am. J. Bot.* 82 1537–1546. 10.2307/2446182

[B63] Young M. D. Wakefield M. J. Smyth G. K. Oshlack A. (2010). Gene ontology analysis for RNA-seq: accounting for selection bias. *Genome Biol.* 11:R14 10.1186/gb-2010-11-2-r14 PMC2872874 20132535

[B64] Yu H. Ito T. Zhao Y. Peng J. Kumar P. Meyerowitz E. M. (2004). Floral homeotic genes are targets of gibberellin signaling in flower development. *Proc. Natl. Acad. Sci. U.S.A.* 101 7827–7832. 10.1073/pnas.0402377101 15128937 PMC419691

[B65] Zhang H. Li A. Zhang Z. Huang Z. Lu P. Zhang D. (2016). Ethylene response factor TERF1, regulated by ETHYLENE-INSENSITIVE3-like factors, functions in reactive oxygen species (ROS) scavenging in tobacco (*Nicotiana tabacum* L.). *Sci. Rep.* 6:29948 10.1038/srep29948 PMC4951782 27435661

[B66] Zhang Y. Liu B. Yang S. An J. Chen C. Zhang X. (2014a). A cucumber DELLA homolog CsGAIP may inhibit staminate development through transcriptional repression of B class floral homeotic genes. *PLoS ONE* 9:e91804 10.1371/journal.pone.0091804 PMC3954735 24632777

[B67] Zhang Y. Zhang X. Liu B. Wang W. Liu X. Chen C. (2014b). A GAMYB homologue CsGAMYB1 regulates sex expression of cucumber via an ethylene-independent pathway. *J. Exp. Bot.* 65 3201–3213. 10.1093/jxb/eru1764 24790111 PMC4071842

[B68] Zhou X. Zhang Z. L. Park J. Tyler L. Yusuke J. Qiu K. (2016). The ERF11 transcription factor promotes internode elongation by activating gibberellin biosynthesis and signaling. *Plant Physiol.* 171 2760–2770. 27255484 10.1104/pp.16.00154 PMC4972265

