# Supplementary material for: Transcriptomic Analysis Implies That GA Regulates Sex Expression via Ethylene-Dependent and Ethylene-Independent Pathways in Cucumber (Cucumis sativus L.)
Source: Front Plant Sci. 2017 Jan 19;8:10. doi: 10.3389/fpls.2017.00010 (PMC5243814; doi:10.3389/fpls.2017.00010)
Supplement: Supplementary file 5 [file Table_5.PDF]

Table S5. List of primers used for qRT-PCR analyses in this study.

Primer	Sequence
SBT33-F	5'-GAGTTGTCCCGAGTTCGCTTTAC-3'
SBT33-R	5'-TTGTTCTCCTGATTCGCATGTTC-3'
CsACS2-F	5'-GAAAACTGTGAGGGAGAAGGGAA-3'
CsACS2-R	5'-GATAGTAAGGAGTGGGGACAAGCA-3'
SCL3-F	5'-TCATGTGGCTGTTGGTAGTCT-3'
SCL3-R	5'-TTCTCAAATGTGGAGGTCCTT-3'
ERF43-F	5'-ACTTCTTTATCTCCCTCTTCCCC-3'
ERF43-R	5'-CAATTATTCCCAATTCATCGTTG-3'
CRF4-F	5'-AATTTCAATCGAGCCATCTTGT-3'
CRF4-R	5'-GCAGTGTCGTAGGTTCCTAACC-3'
SAUR32-F	5'-GTGACCATAGCCAAGAAGAAA-3'
SAUR32-R	5'-CAACTCAACGGAACGACAAAC-3'
WRKY41-F	5'-CCCGATAAGCAAGAACAGAAGG-3'
WRKY41-R	5'-GTCAGGTGAGGTTGGGGAGGTA-3'
CYP450-F	5'-AAGCGAATGCTCAACTTAGGAAG-3'
CYP450-R	5'-GGCAGAGGCAATAGTATCACGAC-3'
MYB21-F	5'-CAAAAAATCAACCAATCCCCGC-3'
MYB21-R	5'-AGTTCAGCATCTCTCCTCCGCC-3'
NAC2-F	5'-CGTCTGTGGTGAAAAGGAGTGG-3'
NAC2-R	5'-TTGTCGTCGGTGGATTGGTAAT-3'
CAG2-F	5'-ATCAGTTAAAGATTTGAAAAGCC-3'
CAG2-R	5'-ACAAGTTGAAGAGAGATAGGGTG-3'
MYB44-F	5'-TGTAGCAAGACGGGAAGCGAAA-3'
MYB44-R	5'-GCTCCAACTCCAAGGAAATCAA-3'
RAP23-F	5'-CACCAGCACTCACTTTCCCGT-3'
RAP23-R	5'-GCACCTCGTTCTCTTCCTCCA-3'
ERF03-F	5'-GTAAGACAAAGGCATTGGGGTT-3'
ERF03-R	5'-TTGCACTTGGACTGTATGGAAA-3'
NAC72-F	5'-ATTCGGGGAAAAGGAATGGTACT-3'
NAC72-R	5'-CTATTGAAGATGAGGGTGAGGCG-3'
SAUR21-F	5'-AAATGAGAAACCAGTCAAATG-3'
SAUR21-R	5'-ATCCAAACTCTTCCTCAACAT-3'
IAA13-F	5'-AAGAGAAGAAGCAAGCAGTAGGA-3'
IAA13-R	5'-TGAGGAGAGCAGTTTTGAGAGAG-3'
CKX1-F	5'-ATTCACTCCCATTAGCTGTTTT-3'
CKX1-R	5'-CGCATTTATTTTCATTTCTGTC-3'
CsETR1-F	5'- GAGACTGAACTTACACCAGAGCA-3'
CsETR1-R	5'- TTTTACAGCATTACCCACAACAT-3'
TUA -F	5'-ACGCTGTTGGTGGTGGTAC-3'
TUA-R	5'-GAGAGGGGTAAACAGTGAATC-3'
